# Prognostic value of DCE-CT-derived blood volume and flow compared to core biopsy microvessel density in patients with metastatic renal cell carcinoma

**DOI:** 10.1186/s41747-021-00232-2

**Published:** 2021-07-30

**Authors:** Aska Drljevic-Nielsen, Finn Rasmussen, Patricia Switten Nielsen, Christina Stilling, Kennet Thorup, Jill Rachel Mains, Hans Henrik Torp Madsen, Frede Donskov

**Affiliations:** 1 Department of Oncology, Aarhus University Hospital (AUH), Palle Juul-Jensens Blvd. 99, 8200 Aarhus N, Denmark; 2 Department of Radiology, Aarhus University Hospital (AUH), Palle Juul-Jensens Blvd. 99, 8200 Aarhus N, Denmark; 3 Department of Pathology, Aarhus University Hospital (AUH), Palle Juul-Jensen Blvd. 99, 8200 Aarhus N, Denmark

**Keywords:** Blood volume, Carcinoma (renal cell), Microvascular density, Prognosis, Tomography (x-ray computed)

## Abstract

**Background:**

Angiogenesis is prominent in metastatic renal cell carcinoma (mRCC). We compared two angiogenesis assessment methods: dynamic contrast-enhanced computed tomography (DCE-CT)-derived blood volume (BV) and blood flow (BF) and core biopsy microvessel density (MVD).

**Methods:**

As planned in DaRenCa Study-1 study, DCE-CT and core biopsy were performed from the same tumour/metastasis at baseline. MVD was assessed by CD34 immunostaining in tumour (CD34-index_T_) or tumour including necrosis (CD34-index_TN_). BV and BF were assessed using the DCE-CT software. Overall survival (OS) and progression-free survival (PFS) were assessed by Kaplan-Meier analysis. Spearman coefficient (rho) tested the correlation between MVD and BV, BF, or CT density (HU).

**Results:**

At baseline, 25 patients had analysable scans and tissue. BV_deconv_, BV_Patlak_, and BF_deconv_ > median were associated with favourable OS (43.2 *versus* 14.6 months, *p* = 0.002; 31.6 *versus* 20.2 months, *p* = 0.015; and 31.6 *versus* 24.5 months, *p* = 0.019). CD34-index_T_ and CD34-index_TN_ did not correlate with age (*p* = 0.543), sex (*p* = 0.225), treatment (*p* = 0.848), International mRCC Database Consortium category (*p* = 0.152), synchronous *versus* metachronous metastatic disease (*p* = 0.378), or tumour volume (*p* = 0.848). CD34-index_T_ or CD34-index_TN_ > median was not associated with PFS (*p* = 0.441 and *p* = 0.854, respectively) or OS (*p* = 0.987 and *p* =0.528, respectively). CD34-index_T_ or CD34-index_TN_ was not correlated with BV, BF, or HU (rho 0.20–0.26).

**Conclusions:**

Differently from MVD, DCE-CT-derived BV and BF had prognostic impact and may better reflect angiogenesis in mRCC.

**Trial registration:**

NCT01274273

## Key points

- Angiogenesis is prominent in patients with metastatic renal cell carcinoma (mRCC).
- Angiogenesis assessed by microvascular density (MVD) was compared to functional imaging.
- Dynamic contrast-enhanced computed tomography-derived functional parameters blood volume (BV) and blood flow (BF) showed strong prognostic ability.
- MVD did not show prognostic potential or association to patient characteristics.
- MVD did not correlate to BV or BF in mRCC.

## Background

Clear cell renal cell carcinoma (ccRCC) is the most frequent kidney cancer histological subtype. Genetic mutations in the Von Hippel Lindau tumour suppressor gene on chromosome three in the proximal convoluted tubule epithelium [1] lead to increased angiogenesis, and thus a highly vascularised tumour [2, 3]. Angiogenesis plays a crucial role in growth, metastasis, and progression in RCC [4]. Therefore, blocking angiogenesis by vascular endothelial growth factor (VEGF) receptor-targeted therapies sorafenib (Nexavar, Bayer, Leverkusen, Germany), sunitinib (Sutent: Pfizer, New York, NY), pazopanib (Votrient: Novartis Oncology, East Hanover, NJ/before 2015: GlaxoSmithKline, Research Triangle Park, NC), tivozanib (Fotivda: EUSA Pharma, Hempstead, UK), cabozantinib (Cabometyx: Ipsen Pharma, Paris, France), or anti-VEGF antibodies such as bevacizumab (Avastin: Genentech, Roche, South San Francisco, CA; all other countries: Basel, Switzerland), has been the mainstay of therapy for more than a decade in patients with metastatic RCC (mRCC) [5]. The disease can also be treated with immunotherapy: interferon-alpha (INF-α2b, IntronA: Merck & Co., Whitehouse Station, NJ) and interleukin-2 (aldesleukin, Proleukin: Novartis Vaccines and Diagnostics, East Hanover, NJ) were first shown to be effective [6] followed by the checkpoint inhibitors nivolumab (Opdivo: Bristol Myers Squibb, New York, NY) and ipilimumab (Yervoy: Bristol Myers Squibb, New York, NY) [7]. Recently, immunotherapy combined with VEGF inhibitors has shown promising activity [8–11].

Dynamic contrast-enhanced computed tomography (DCE-CT) is a non-invasive functional imaging method that can assess angiogenesis. The method consists of repeated computed tomography (CT) scans of a tumour and can analyse changes in contrast enhancement in blood vessels and tissue over time. This enables an assessment of vascular support over the scanned tissue by performing a calculation of functional perfusion parameters such as blood flow (BF) and blood volume (BV), parameters which are closely correlated with angiogenesis [12–14]. In patients with mRCC, high baseline BV has been associated with favourable baseline clinical factors, while low BV has been associated with unfavourable baseline clinical factors, and high baseline BV has been demonstrated as an independent favorable prognostic factor for survival outcome [15]. Furthermore, a decline in DCE-CT-identified parameters BV or BF during early treatment has shown correlation to favourable survival outcomes [16–18].

The degree of angiogenesis in a tumour can also be histologically characterised by the microvessel density (MVD) technique, where immunohistochemistry (IHC) staining is used to stain blood vessels [19]. Tumour MVD as a prognostic factor has been reported in several cancers, with high vascularity being associated with poorer patient outcomes in breast, ovarian, and prostate cancers [20–22]. In primary RCC, MVD as a prognostic factor for survival outcome has shown conflicting results [23–26], and there is limited knowledge about MVD as a prognostic factor in mRCC. In patients with primary RCC [27, 28], assessment of angiogenesis using DCE-CT and MVD showed a positive correlation, whereas such correlation has not been assessed in patients with mRCC.

The primary aim of this study was to compare two different methods for assessing angiogenesis in the same tumour lesion at baseline in patients with mRCC: DCE-CT parameters BV and BF compared with MVD. The secondary aim was to assess the prognostic ability of BV, BF, and MVD.

## Methods

### Patients and treatment

Patients with biopsy-verified mRCC were included in a prospective randomised phase II clinical trial, the Danish Renal Cancer Group Study-1 (DaRenCa-1), at Aarhus University Hospital, Denmark, from October 2009 to November 2014. Patients (*n*= 89) were randomised to receive subcutaneously administered interleukin 2 (IL-2) and interferon alpha (INF-α) with or without intravenously administered bevacizumab (*n*= 45 and *n*= 44, respectively). Functional imaging using DCE-CT and a corresponding core biopsy from the same tumour lesion was an integrated part of the study. All included patients were offered a biopsy, if safe and technically feasible at baseline.

Before entering the study, informed consent was obtained from all patients. The study was approved by the Danish Medicines Agency (journal no. 2612-4042), the Regional Ethics Committee (case no. 1-10-72-472-12), and the Danish Data Protection Agency (journal no. 2012-41-0897). Darenca-1 was registered at ClinicalTrials.gov (identifier NCT01274273).

The clinical results from DaRenCa-1 [8], the prognostic impact on survival outcome of baseline DCE-CT identified BV [15], and the association between DCE-CT parameters and early treatment response [16–18] in this patient group have previously been published.

Electronic medical records were used to obtain baseline patient characteristics such as age, nephrectomy status, tumour histology, and the International Metastatic Renal Cell Carcinoma Database Consortium (IMDC) risk group factors [29].

### CT technique

At baseline, DCE-CT and routine contrast-enhanced CT scans were performed using Philips Brilliance 64 or iCT 256 (Philips Healthcare, Best, The Netherlands). Firstly, a DCE-CT scan over a single target lesion was performed preceded by intravenous administration of 60 mL iodixanol (Visipaque, General Electric Healthcare, Princeton, NJ, USA) 270 mg I/mL at 6 mL/s. The DCE-CT scans comprised of 2-s scan cycles (total 70 s), median 100 kVp (range, 80–120 kVp), median *z*-axis 8 cm (range, 4–13.5 cm), and median 100 mAs (range, 100–210 mAs). The median CT dose index was 131.51 mGy (range, 28.80–184.50 mGy). The median dose length product was 1,052.10 mGy × cm (range, 434.80–27,45.50 mGy × cm). Secondly, the patients remained supine for 10 min before performing a routine contrast-enhanced CT of the thorax, abdomen, and pelvis preceded by administering intravenous iodixanol (Visipaque, GE Healthcare, Princeton, NJ) 270 mg I/mL based on body weight (maximum 180 mL) at 4 mL/s. In case of a previous minor reaction to iodixanol, administration of iohexol (Omnipaque, GE Healthcare, Princeton, NJ) 300 mg I/mL was used. Contrast-enhanced CT was performed using 120 kVp of peak voltage, 0.75 s of rotation time, a pitch of 0.925, a collimation of 64 or 128 × 0.625 mm, and attenuation-based current modulation. Prior to the administration of intravenous contrast agent media, all patients were given 1-L saline solution intravenously to ensure hydration.

Routine contrast-enhanced CT scans were performed every third month until progressive disease and assessed according to the criteria laid out in Response Evaluation Criteria in Solid Tumors, version 1.1 (RECIST v.1.1) [30, 31] by experienced radiologists and used for clinical purpose, whereas DCE-CT scans were used exclusively for research purpose.

### Image analysis

DCE-CT data were analysed in four dimensions using the Advanced Perfusion and Permeability Application (APPA) (Philips, Healthcare, Best, The Netherlands).

After loading the dynamic data in the APPA software program, a non-rigid registration used for motion correction and spatial filtration was performed. The dynamic data were processed and analysed at arterial peak enhancement. DCE-CT parameters BV deconvolution (BV_deconv_, mL × 100 g^−1^) and BF deconvolution (BF_deconv_, mL × 100 g^−1^/min) were calculated using the deconvolution method; BV Patlak (BV_Patlak,_ mL × 100 g^−1^) was calculated using the two-compartment method [13]. The corresponding BV and BF series were displayed together with the morphologic series at arterial peak enhancement.

DCE-CT data were loaded into Intellispace 6.0 Multimodality Tumor Tracking, (Philips, Healthcare, Best, The Netherlands), where the entire target lesion was segmented on the morphologic series at arterial peak enhancement using a three-dimensional semiautomatic sculpt tool and was defined as the volume of interest (VOI). A blinded radiologist with 2 years of training in DCE-CT performed all the analyses (A.D.N.). An excellent interobserver correlation in the analyses of functional imaging CT data in this patient group has previously been found [18].

Dynamic data were loaded and analysed in MATLAB (v. R2015b, MathWorks Inc., Natick, MA, USA) based on the delineated VOI at arterial peak enhancement using an in-house software.

The median values for the DCE-CT parameters were calculated using histogram analysis. We used the median values of BV_deconv_, BV_Patlak_, and BF_deconv_, as these three parameters have shown the best correlation with survival outcome in patients with mRCC [18]. In addition, the contrast-enhanced tissue density measured as Hounsfield units (HU) was assessed for each target lesion at arterial peak enhancement.

### Biopsies

According to prespecified protocol criteria, experienced radiologists (H.H.T.M. or J.M.) defined either a primary tumour or metastasis as a target lesion at baseline, suitable for core biopsy and functional imaging using CT [32].

All core biopsies were performed by an experienced radiologist under ultrasound guidance either the same day as the DCE-CT and contrast-enhanced CT scan or the subsequent day. As a standard, two core biopsy samples were taken from each target lesion using an 18-G automated core biopsy needle. As a default, the same target lesion was used for DCE-CT and core biopsy, *i.e.*, matched target lesions. However, this was not always technically possible for all target lesions. Out of the 89 included patients, a total of 43 patients had a biopsy performed at baseline. Unmatched biopsies (*n* = 9) and biopsies without representative tumour specimen (*n* = 9) were excluded from the study. A total of 25 patients with a matched target lesion were included in the final analysis (Table 1).

**Table 1:** Target lesion localisation

	All patients (*n* = 25)	Patients with synchronous metastases (*n* = 19)	Patients with metachronous metastases (*n* = 6)
Primary kidney tumour	4	4	-
Liver	4	2	2
Lung/pleura	4	4	-
Lymph node	2	2	-
Kidney (metastases)	2	1	1
Retroperitoneum	4	2	2
Bone (soft tissue component)	5	4	1

### IHC staining and MVD

The core biopsy specimens were fixed with 10% formaldehyde and paraffin-embedded. Each paraffin block was cut into 3-μm-thick serial slices and mounted on glass slides. From each paraffin block, one slide was stained with hematoxylin and eosin (Ventana HE 600, Ventana Medical Systems, Tucson, AZ, USA) to identify tumour regions, and one slide was stained with monoclonal mouse antibody CD34 (QBEnd-10; Ventana, as above) by Benchmark XT (Ventana) to assess MVD. CD34 was chosen to quantify the vessels in the tumour, as this method has been shown to be specific and reproducible for this purpose [33, 34]. Standard settings and reagent kits were used in deparaffinisation, rehydration, antigen retrieval, and endogenous peroxidase blocking. CD34 was visualised by OptiView 3,3′-Diaminobenzidine IHC Detection Kit (Ventana, as above). Using Mayer’s hematoxylin and bluing reagent, all slides were counterstained. Both slides were digitalised at 20× by NanoZoomer 2.0HT (Hamamatsu Photonics K.K., Hamamatsu City, Japan) and superimposed to form a virtual stain in the Visiopharm Integrator System 2019.02.1.6005 (Visiopharm A/S, Hørsholm, Denmark).

An experienced uropathologist (C.S.) with 10 years of pathology training manually outlined tumour regions and necrosis regions on the digitised HE slides in Visiopharm; these outlines were automatically transferred to the CD34 slides within the virtual slide. Based on a threshold of the CD34 staining colour, marked by a colour deconvolution step, an automated quantification of CD34 was obtained. To exclude inaccuracies of the automated detection, all analyses were also reviewed manually by P.S.N., with 10 years of training in digital pathology. MVD was assessed using the CD34-index in both the tumour outline (T), defined as the CD34-index_T_, and in the combined tumour and necrosis outline (TN), defined as CD34-index_TN_, respectively, and was calculated using the following equation:

$$ \frac{\mathrm{CD}34\ \mathrm{positive}\ \mathrm{T}\ \mathrm{or}\ \mathrm{T}\mathrm{N}\ \mathrm{area}}{\mathrm{Total}\ \mathrm{T}\ \mathrm{or}\ \mathrm{T}\mathrm{N}\ \mathrm{area}}\ \mathrm{x}\ 100\% $$

### Statistical analysis

Progression-free survival (PFS) was defined as the period between study inclusion and RECIST v1.1-defined progression/cancer-related death. The period between inclusion and death was defined as the overall survival (OS). Kaplan-Meier survival curves and log-rank tests were used to test the association between baseline CD34-index as a categorical variable, using the median value as a cutoff, (> *versus* ≤ median) and survival outcome. The association between CD34-index as a continuous variable and survival outcome was tested using Cox regression analysis. The association between baseline CD34-index and baseline clinical factors, age, gender, and treatment group, was tested using χ^2^ test or Fischer exact test, when applicable. Synchronous metastatic disease is defined as presence of metastases ≤ 3 months of initial cancer diagnosis, metachronous metastatic disease as presence of metastases > 3 months of initial diagnosis.

The median follow-up time in alive patients was assessed using the reverse Kaplan-Meier survival method. The association between baseline clinical factors in this present study (*n* = 25) was compared to baseline clinical factors in the previously published study (*n* = 105) [15] using χ^2^ or Fischer exact test, when applicable.

DCE-CT parameters and CD34-index had a clear non-Gaussian distribution assessed visually on probability histograms. Therefore, non-parametric Spearman’s rank correlation coefficient, rho, was used to test the correlation between DCE-CT parameters or HU and the CD34-index in the tumour outline and the combination of tumour and necrosis outline, respectively; *p-*values < 0.05 were regarded as statistically significant. All statistical tests were two-sided.

All statistical analyses were performed using IBM SPSS Statistics for Windows (Version 27.0, IBM Corp., Armonk, NY, USA).

## Results

### Patient characteristics

At baseline, 25 patients had analysable scans and tissue (Table 1). All patients had clear cell tumour histology. The median age was 57.2 years (range 37.5–66.4), 18 (72%) patients were male, and 19 (76%) patients had synchronous metastatic disease. According to the IMDC classification, two (8%) patients had favourable, 18 (72%) patients had intermediate, and five (20%) patients had poor prognosis. A total of 13 (52%) patients were treated with IL-2 and IFN-α, and 12 (48%) patients were treated with IL-2, IFN-α, and bevacizumab. The median volume of the target lesions was 44.9 cm^3^ (range 4.79–427.45). The median follow-up time in alive patients was 112.3 months, the median PFS was 6.3 months (range 0.5–118.6), and the median OS was 27.8 months (range 0.9–125.0).

To control for imbalances in established prognostic factors, we compared baseline patient characteristics in the present cohort (*n* = 25) with the previously published cohort (*n* = 105) [15]. We found no difference in IMDC risk groups (*p* = 0.188), sex (*p* = 0.893), treatment (*p* = 0.122), age (*p* = 0.371), synchronous *versus*. metachronous disease (*p* = 0.083), or target lesion volume (*p* = 0.589).

### MVD and DCE-CT parameters

The CD34-index_T_ and CD34-index_TN_ at baseline were 6.76% (range 0.78–19.23) and 6.15% (range 0.78–19.23), respectively. Median BV_deconv_ was 29.00 mL × 100 g^−1^ (range 11.41–70.03), BV_Patlak_ was 20.45 mL × 100 g^−1^ (range 3.69–68.64), BF_deconv_ was 159.88 mL × 100 g^−1^/min (range 52.33–346.24), and HU was 89.00 (range 50.00–231.00).

**Fig. 1:**
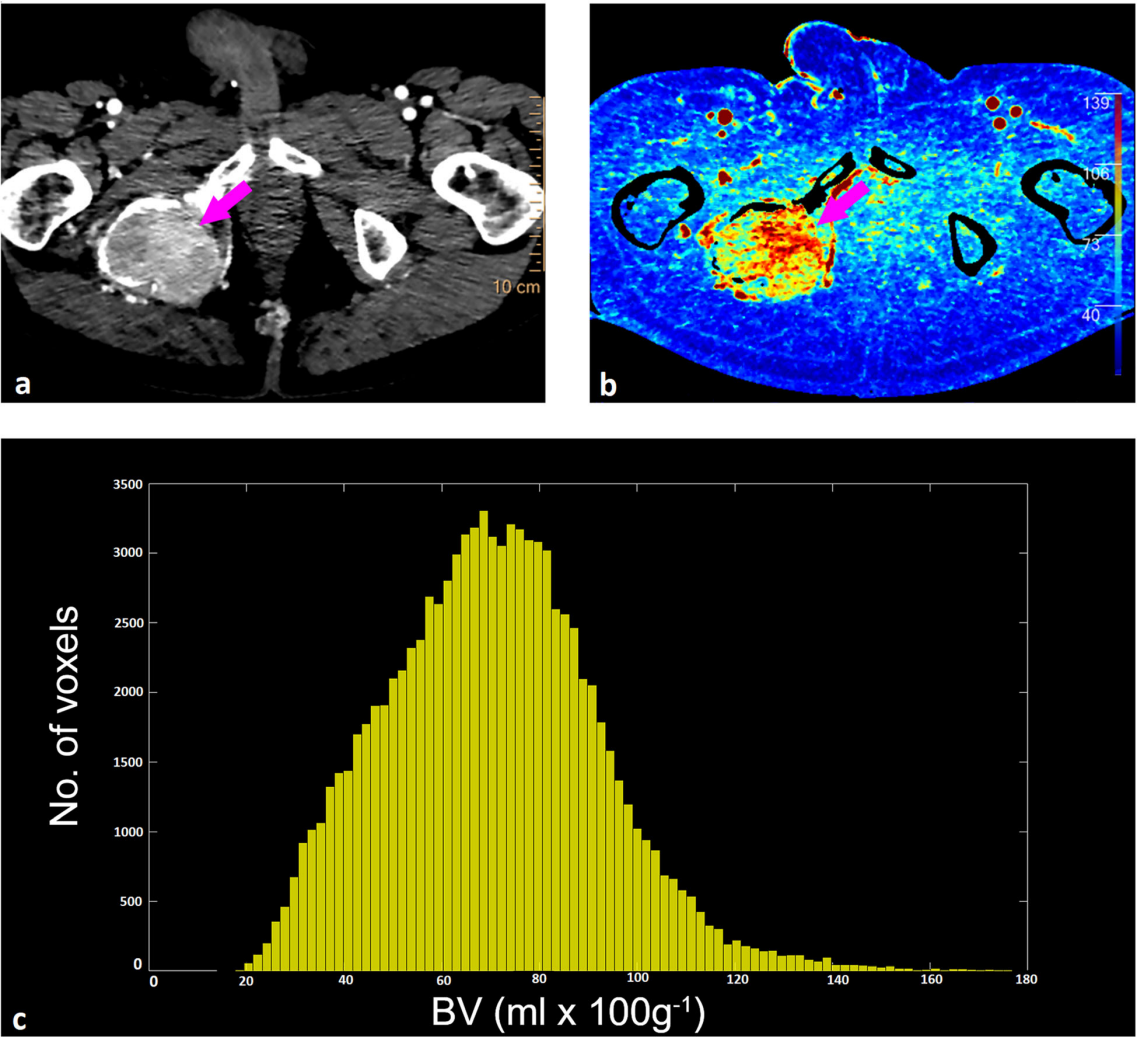
A heterogeneous metastasis in the right iliac bone (purple arrow) at baseline presented on **(a)** CE-CT, **(b)** BV_deconv_ map, and **(c)** the corresponding BV_deconv_ histogram. The histogram depicts a large BV range, illustrating the intratumoural heterogeneity. *BV*_*deconv*_ Blood volume (deconvolution), *CE-CT* Contrast-enhanced computed tomography, *No.* Number

The histogram width (*i.e.*, the difference between the minimum and maximum values of the histograms) at baseline for BV_deconv_ was 90.69 mL × 100 g^−1^ (range 24.38–618.21), for BV_Patlak_ was 68.28 mL × 100 g^−1^ (range 23.83–381.85), for BF_deconv_ was 511.42 mL × 100 g^−1^/min (range 86.67–3,640.46), and for the tissue density was 366 HU (range 75.00–1,882.00) (Fig. 1).

**Fig. 2:**
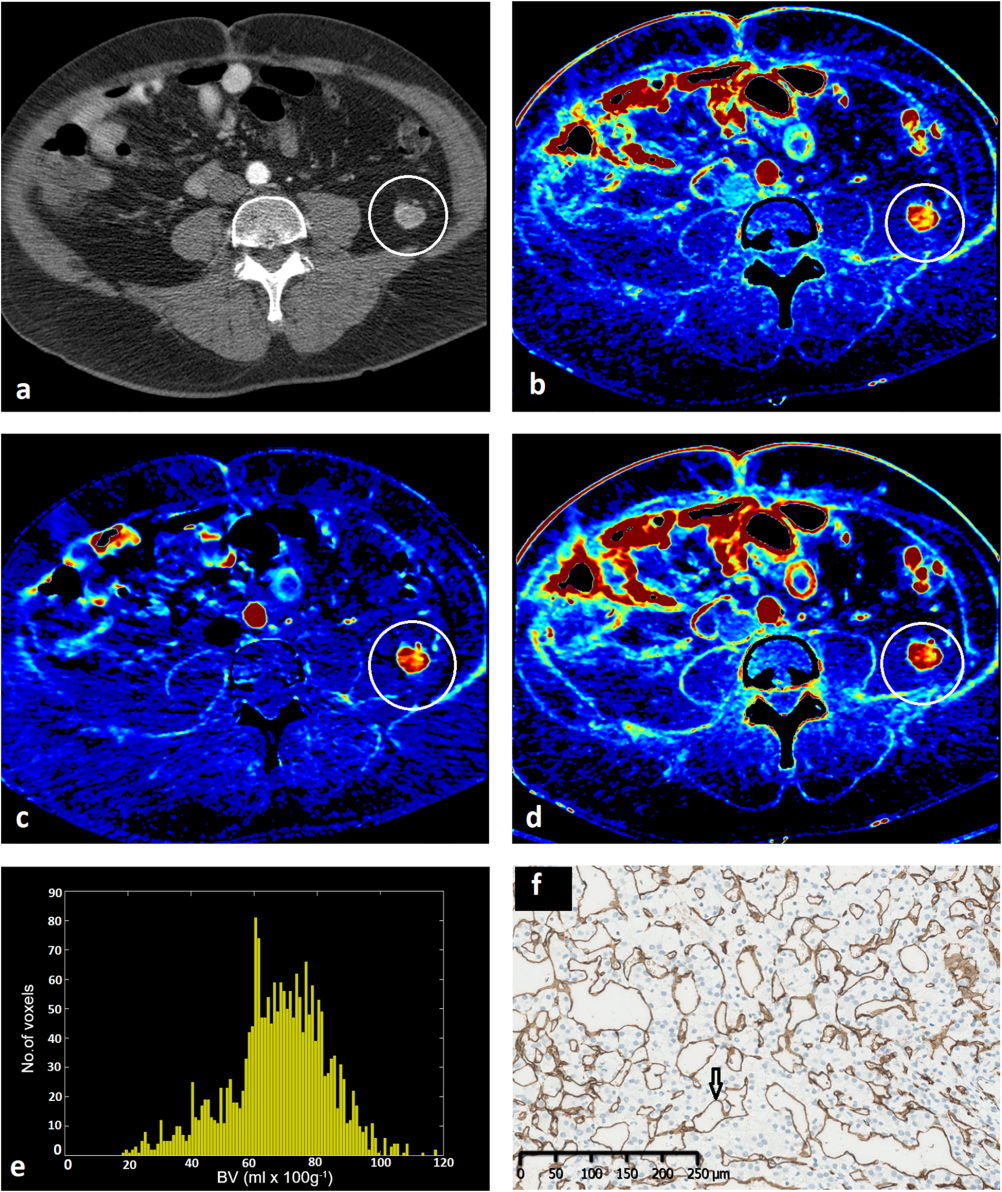
A retroperitoneal metastasis marked with a white circle depicted on **(a)** a contrast-enhanced CT at peak arterial enhancement with the corresponding functional imaging colour maps, **(b)** BV_deconv_, **(c)** BV_Patlak_, and **(d)** BF_deconv_. BV_deconv_ histogram for the entire target lesion at baseline is shown in **e**, while **f** represents the CD34 immunostaining from the same retroperitoneal metastasis with microvessels (arrow) stained brown. *BV*_*deconv*_ Blood volume (deconvolution), *BV*_*Patlak*_ Blood volume (Patlak), *BF*_*deconv*_ Blood flow (deconvolution)

An example of CE-CT, DCE-CT, and CD34 IHC staining from the same target lesion in a representative patient is illustrated in Fig. 2.

### MVD and baseline characteristics

No statistically significant association was found between CD34-index_TN_ and age (*p* = 0.543), sex (*p* = 0.748), treatment group (*p* = 0.848), IMDC prognostic group (*p* = 0.152), target lesion volume (*p* = 0.434), or synchronous *versus*. metachronous metastatic disease (*p* = 0.378). Similarly, no association was found between CD34-index_T_ and baseline patient characteristics (Table 2).

**Table 2:** Baseline patient characteristics

	CD34-index_T_		
	Above median (>), *n* (%)	Below median (≤), *n* (%)	*p*-value
Total	12	13	
Age (years)			0.543
> Median (57.2)	5 (41.7)	7 (58.3)	
≤ Median (57.2)	7 (53.8)	6 (46.2)	
Sex			0.225
Male	10 (55.6)	8 (44.4)	
Female	2 (28.6)	5 (71.4)	
Treatment group			0.848
IL2/ IFN-α	6 (46.2)	7 (53.8)	
IL2/INF-α/bevacizumab	6 (50.0)	6 (50.0)	
IMDC category			0.152
Favourable	2 (100.0)	-	
Intermediate	9 (50.0)	9 (50.0)	
Poor	1 (20.0)	4 (80.0)	
Target lesion volume (cm^3^)			0.848
> Median (44.9)	6 (46.2)	7 (53.8)	
≤ Median (44.9)	6 (50.0)	6 (50.0)	
Synchronous metastases			0.378
Yes	8 (42.1)	11 (57.9)	
No	4 (66.7)	2 (33.3)	

*CD34-index*_*T*_ CD34-index in the tumour outline, *IMDC* International Metastatic Renal Cell Carcinoma Database Consortium, *IFN-α* Interferon alpha, *IL-2* Interleukin-2

### MVD, DCE-CT, and survival outcome

**Fig. 3:**
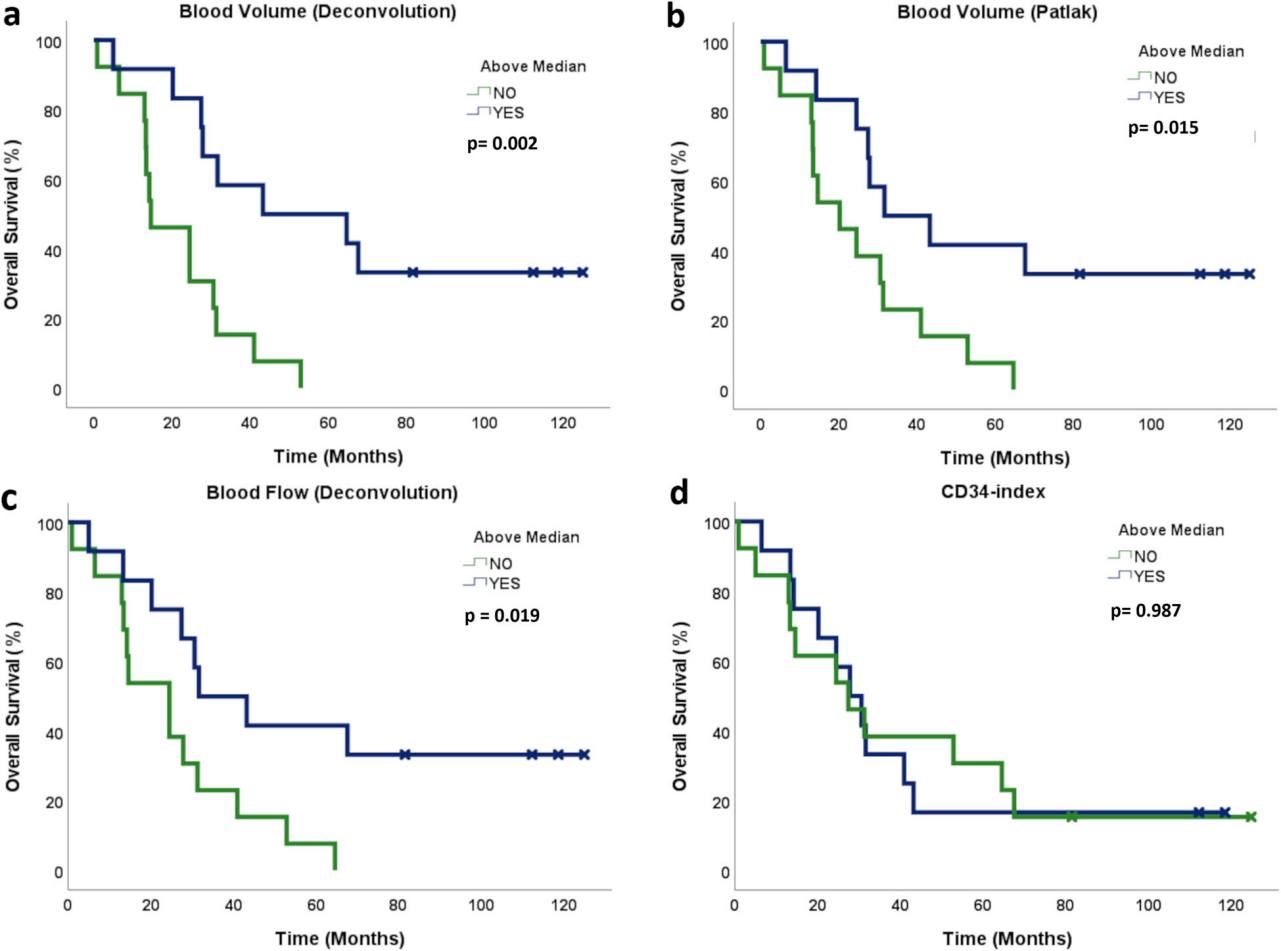
Kaplan-Meier curves for overall survival for high *versus* low **(a)** BV_deconv_, **(b)** BV_Patlak_, **(c)** BF_deconv_, and **(d)** CD34-index in tumor, respectively, demonstrating favourable survival outcome for high BV_deconv_, BV_Patlak_, and BF_deconv_, whereas CD34-index was not associated to survival outcome. *BV*_*deconv*_ Blood volume (deconvolution), *BV*_*Patlak*_ Blood volume (Patlak), *BF*_*deconv*_ Blood flow (deconvolution)

CD34-index_T_ and CD34-index_TN_ > median were not associated with survival outcome, with similar PFS (6.7 *versus* 5.6 months; *p* = 0.441 and *p* = 0.854, respectively) and near-identical OS (27.8 *versus* 27.4 months; *p* = 0.987 and *p* = 0.528, respectively). DCE-CT parameters BV_deconv_, BV_Patlak_, and BF_deconv_ > median were all associated with favourable OS (43.2 *versus* 14.6 months, *p* = 0.002; 31.6 *versus* 20.2, *p* = 0.015; and 31.6 *versus* 24.5 months, *p* = 0.019, respectively), but not with PFS (5.3 *versus* 6.3 months, *p* = 0.411; 6.7 *versus* 5.3 months, *p* = 0.317; and 5.3 *versus* 6.3, *p* = 0.531, respectively) (Fig. 3).

Assessing baseline CD34-index_T_ and CD34-index_TN_ as a continuous variable also showed no correlation with PFS (hazard ratio (HR) 0.99, 95% confidence interval (CI) 0.93–1.06, *p* = 0.824 and HR 1.00, 95% CI 0.94–1.07, *p* = 0.905) or OS (HR 1.00, 95% CI 0.93, 1.08, *p* = 0.930 and HR 1.01; 95% CI 0.95, 1.08, *p* = 0.684), respectively. Adjusting baseline CD34-index_T_ and CD34-index_TN_ for treatment group, there still was no correlation with PFS (HR 1.03, 95% CI 0.96–1.11, *p=* 0.404 and HR 1.04, 95% CI 0.97–1.11, *p=* 0.288) and OS (HR 1.00, 95% CI 0.93–1.08, *p=* 0.936 and HR 1.02, 95% CI 0.95–1.09, *p=* 0.677), respectively.

### Correlation between MVD and DCE-CT parameters

**Fig. 4:**
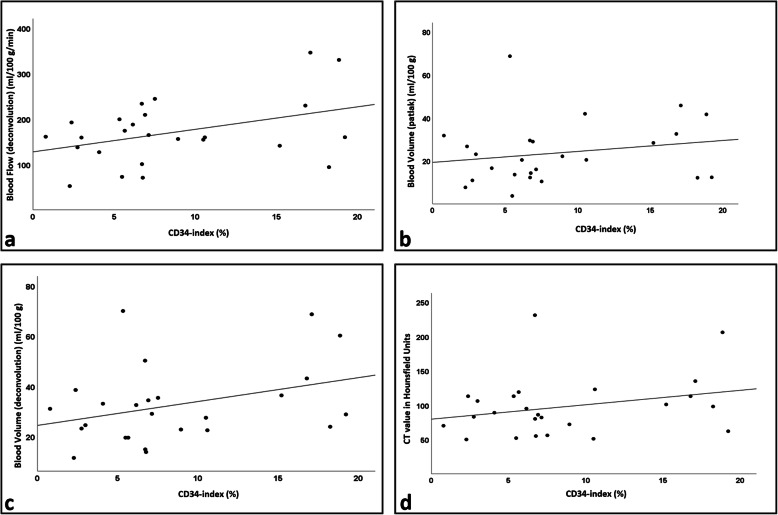
Plots illustrating no correlation between CD34-index in tumor outlines and **(a)** BF_deconv_, **(b)** BV_Patlak_, **(c)** BV_deconv_, and **(d)** HU, respectively at baseline. *BV*_*deconv*_ Blood volume (deconvolution), *BV*_*Patlak*_ Blood volume (Patlak), *BF*_*deconv*_ Blood flow (deconvolution), *HU* Hounsfield units

No statistically significant correlation was found between CD34-index_T_ or CD34-index_TN_ and DCE-CT-derived parameters BV and BF at baseline (Table 3 and Fig. 4). This lack of correlation remained when stratifying patients for synchronous *versus* metachronous mRCC (Table 3). Furthermore, there was no correlation between HU and CD34-index_T_ or CD34-index_TN_, respectively (Table 3 and Fig. 4).

**Table 3:** Correlations between DCE-CT parameters, HU, and CD34-index at baseline

	All patients (*n* = 25) SCC (*p*-value)		Synchronous mRCC (*n* = 19) SCC (*p*-value)		Metachronous mRCC (*n* = 6) SCC (*p*-value)	
	CD34-index_T_	CD34-index_TN_	CD34-index_T_	CD34-index_TN_	CD34-index_T_	CD34-index_TN_
BV_deconv_	0.23 (0.275)	0.21 (0.317)	0.20 (0.416)	0.19 (0.429)	0.14 (0.787)	0.14 (0.787)
BV_Patlak_	0.20 (0.351)	0.17 (0.417)	0.23 (0.336)	0.22 (0.367)	0.09 (0.872)	0.09 (0.872)
BF_deconv_	0.26 (0.207)	0.22 (0.289)	0.27 (0.273)	0.23 (0.344)	0.14 (0.787)	0.14 (0.787)
HU	0.20 (0.351)	0.22 (0.285)	0.12 (0.624)	0.18 (0.466)	0.31 (0.544)	0.31 (0.544)

*BV*_*deconv*_ Blood volume (deconvolution), *BV*_*Patlak*_ Blood volume (Patlak), *BF*_*deconv*_ Blood flow (deconvolution), *CD34-index*_*TN*_ CD34-index in the tumour necrosis outline, *CD34-index*_*T*_ CD34-index in the tumour outline, *DCE-CT* Dynamic contrast-enhanced computed tomography, *HU* Hounsfield units, *mRCC* Metastatic renal cell carcinoma, *SCC* Spearman correlation coefficient (rho)

## Discussion

To our knowledge, this study is the first to assess two different methods in the evaluation of angiogenesis in patients with mRCC. At baseline from the same tumour lesion, a core biopsy was obtained to assess MVD by immunohistochemistry, in parallel with a DCE-CT scan to calculate BV and BF. We identified BV and BF to have strong prognostic ability; patients with high BV or BF had favourable survival. In contrast, MVD did not show prognostic potential, and moreover, MVD did not correlate to baseline patient characteristics, to DCE-CT parameters BV or BF, or to HU. Despite only 25 patients had evaluable scans and tumours, this study represents the largest cohort of patients with metastatic renal cell carcinoma examined to date with an integrated prospectively obtained functional imaging CT and corresponding biopsy design [8]; all analyses were preplanned per protocol.

No correlations between DCE-CT parameters BV and BF and MVD were found regardless of assessing the CD34-index in isolated tumours or tumours including necrosis. This lack of correlation remained after stratification of patients into those with synchronous mRCC and metachronous mRCC. Although, no adjustments for multiple testing were performed in this study, there was no statistically significant correlation, which further highlighted the lack of correlation. Furthermore, baseline MVD was not associated with baseline patient characteristics, HU, or with survival outcome independent of treatment group, thus indicating that baseline MVD does not have prognostic impact in mRCC. However, further research evaluating this matter in a larger cohort is encouraged, including evaluating the correlation between DCE-CT parameters and MVD during ongoing oncologic treatment.

Two previous studies have found a positive correlation between DCE-CT-identified parameters and MVD assessed by CD34 IHC in primary RCC undergoing nephrectomy [27, 28]. Chen et al. [27] found a positive correlation between MVD and DCE-CT-identified BV, BF, and permeability surface area product in 77 patients that had undergone surgical resection for primary RCC. In that study, the DCE-CT parameters were assessed using one circular ROI on a 5-mm tumour section, and MVD was assessed in the surgical specimen sampled at the level corresponding to the DCE-CT assessment. Wang et al. also found a positive correlation between DCE-CT parameters and MVD in 24 patients with primary RCC undergoing surgical resection [28]. In that study, DCE-CT scans were assessed using either one or two circular ROIs, while MVD was assessed from the surgical specimen sampled corresponding to the ROIs selected at DCE-CT [28]. Employing this method, Chen et al. [27] and Wang et al. [28] assessed the DCE-CT parameters and MVD in the exactly same region of the tumour and found a close correlation between BV or BF and MVD. In contrast, the DCE-CT-identified parameters in our study were assessed using histogram analysis of the entire target lesion, mostly metastatic lesions, using a VOI, whereas MVD was assessed from a single core biopsy; this could explain the difference in our results compared to the results of Wang et al. [28] and Chen et al. [27]. Consistent with our findings, a study by Puerto-Nevado et al. [35] in patients with mRCC found no prognostic impact of MVD assessed at metastatic sites in 23 patients treated with sunitinib.

Recently, the evolutionary trajectories of kidney cancer were described in more detail by the TRACERx Renal study, and the authors identified large intratumoural heterogeneity in mRCC [36]. The results from our study indicated that angiogenesis assessed by a single core biopsy from a target lesion may not reflect the angiogenesis of the entire target lesion and support the findings of intratumoral heterogeneity in mRCC [36]. The great width of the histograms (*i.e*., the large difference between the minimum and maximum values of the histogram) in our assessments also reflects the intratumoural heterogeneity. Furthermore, heterogeneity between patients with synchronous and metachronous mRCC has been shown [37, 38]. However, in our study, we found no difference between baseline MVD in patients with synchronous *versus* metachronous mRCC, and the lack of correlation between MVD and DCE-CT parameters remained after analysing patients with synchronous and metachronous mRCC separately.

We have previously established that the DCE-CT parameter BV was an independent prognostic factor that added to the prognostic accuracy of IMDC [15–18]. Analogous to our previous findings, we were able to identify the same positive association between overall survival and baseline BV_deconv_, BV_Patlak_, or BF_deconv_ in only 25 patients in the present study. This finding highlights the robustness and strength of DCE-CT parameters BV and BF as prognostic factors. Furthermore, we found no difference in baseline patient characteristics between patients from this study (*n* = 25) and the previous study (*n* = 105), thereby excluding the risk of sampling bias.

Assessment of angiogenesis using MVD has several disadvantages: it is an invasive procedure that is associated with greater discomfort and risk of procedure complications, it does not allow repeated measurements, and it does not take account for the intratumoural heterogeneity [27, 36]. However, assessment of angiogenesis can be performed noninvasively using functional imaging DCE-CT scan method; by histogram analysis of the entire tumour lesion, this method takes account for intratumoural heterogeneity [12–14]. In contrast, a single core biopsy only provides information from a small fraction of the tumour [36]. The non-invasive DCE-CT method may be more suitable in the assessment of angiogenesis in patients with mRCC.

The study was limited by the low number of patients. A limitation to DCE-CT is motion artefacts due to the repeated scans. However, the motion artefacts were minor and could be corrected using the non-rigid registration feature in the APPA software program, which resulted in no patients being excluded due to motion artefacts. Another limitation to the DCE-CT technique is the high radiation dose. However, due to the reduced life expectancy in mRCC, the risk of radiation-induced cancer is considered to be relatively low.

In conclusion, DCE-CT parameters BV and BF had a prognostic impact and may better reflect angiogenesis in mRCC. However, validation in a larger study is encouraged.

## Data Availability

The datasets generated and/or analyzed during the current study are not publicly available due to further analysis of data for upcoming publications, but are available from the corresponding author on reasonable request.

## Abbreviations

APPA: Advanced perfusion and permeability application
BF: Blood flow
BV: Blood volume
BF_deconv_: BF deconvolution
CI: Confidence interval
CT: Computed tomography
DaRenCa-1: Danish Renal Cancer Group Study-1
DCE-CT: Dynamic contrast-enhanced CT
HR: Hazard ratio
IHC: Immunohistochemistry
IL-2: Interleukin 2
IMDC: International Metastatic Renal Cell Carcinoma Database Consortium
INF-α: Interferon alpha
mRCC: Metastatic renal cell carcinoma
MVD: Microvessel density
OS: Overall survival
PFS: Progression-free survival
RECIST v1.1: Response Evaluation Criteria in Solid Tumors, version 1.1
T: Tumour outline
TN: Tumour and Necrosis outline
VEGF: Vascular endothelial growth factor
VOI: Volume of interest
